# Electricity-Free Amplification and Detection for Molecular Point-of-Care Diagnosis of HIV-1

**DOI:** 10.1371/journal.pone.0113693

**Published:** 2014-11-26

**Authors:** Jered Singleton, Jennifer L. Osborn, Lorraine Lillis, Kenneth Hawkins, Dylan Guelig, Will Price, Rachel Johns, Kelly Ebels, David Boyle, Bernhard Weigl, Paul LaBarre

**Affiliations:** 1 PATH, Seattle, Washington, United States of America; 2 Oregon Health and Science University School of Medicine, Portland, Oregon, United States of America; New York University, United States of America

## Abstract

In resource-limited settings, the lack of decentralized molecular diagnostic testing and sparse access to centralized medical facilities can present a critical barrier to timely diagnosis, treatment, and subsequent control and elimination of infectious diseases. Isothermal nucleic acid amplification methods, including reverse transcription loop-mediated isothermal amplification (RT-LAMP), are well-suited for decentralized point-of-care molecular testing in minimal infrastructure laboratories since they significantly reduce the complexity of equipment and power requirements. Despite reduced complexity, however, there is still a need for a constant heat source to enable isothermal nucleic acid amplification. This requirement poses significant challenges for laboratories in developing countries where electricity is often unreliable or unavailable. To address this need, we previously developed a low-cost, electricity-free heater using an exothermic reaction thermally coupled with a phase change material. This heater achieved acceptable performance, but exhibited considerable variability. Furthermore, as an enabling technology, the heater was an incomplete diagnostic solution. Here we describe a more precise, affordable, and robust heater design with thermal standard deviation <0.5°C at operating temperature, a cost of approximately US$.06 per test for heater reaction materials, and an ambient temperature operating range from 16°C to 30°C. We also pair the heater with nucleic acid lateral flow (NALF)-detection for a visual readout. To further illustrate the utility of the electricity-free heater and NALF-detection platform, we demonstrate sensitive and repeatable detection of HIV-1 with a ß-actin positive internal amplification control from processed sample to result in less than 80 minutes. Together, these elements are building blocks for an electricity-free platform capable of isothermal amplification and detection of a variety of pathogens.

## Introduction

Nucleic acid amplification tests (NAATs) are very effective for diagnosing the infectious diseases that affect global health. However, their use in low-resource settings (LRS) has been extremely limited in part due to the requirements for complex instrumentation. Most commercially available NAATs require reliable electricity, expensive equipment, cold storage for reagents, temperature-controlled environments, and highly trained technicians. Most NAAT-capable laboratories in LRS are located in urban areas and cater primarily to the affluent or are used for high-throughput screening of specimens shipped in from other regions. Specimen shipping and laboratory backlogs can delay timely reporting of test results back to the clinic and increase loss to follow-up [1]–[5]. In contrast, rural health care facilities may enable faster results but commonly have only basic equipment [6]. Health care workers in these settings have limited training and are unable to maintain equipment and reliably handle reagents. Mains electrical power, when available, is often unreliable [7]. Despite multiple attempts to develop portable, low-cost, simplified NAATs that can be used in LRS, few are commercially available and none are in widespread use [8].

The most commonly used NAAT method, the polymerase chain reaction (PCR), is not ideal for LRS using current commercially available equipment. Traditional PCR-based diagnostics require a thermocycler, a clean laboratory, reliable electricity, cold storage for reagents, a temperature-controlled environment, provisions for amplicon containment, and trained personnel. Many PCR machines are controlled via a computer, which increases purchase cost.

Recently, there have been significant developments in isothermal amplification methods that do not require thermocycling [9]–[13]. Among these, loop-mediated isothermal amplification (LAMP) is one of the most published methods [14]. A search of the ISI-Web of Knowledge database using the search terms ‘LAMP’ and ‘loop mediated amplification’ returns 1,230 publications since the first description of the method in 2000. LAMP can be used for the amplification of DNA, and when a reverse transcription step is included, LAMP can also amplify from RNA [15]. LAMP is sufficiently sensitive for clinical use [16] and is much less susceptible to inhibitors than PCR [17]. Amplification occurs at one constant temperature, typically in a range between 58°C and 65°C [9], [18]. Set-up is relatively simple, and direct turbidity or fluorescence detection is possible, [17] although other naked-eye-detection schemes such as visualization on an immunochromatographic strip have also been evaluated [19]. Furthermore, the complex sample preparation steps required for PCR can be simplified or eliminated with LAMP [20]. Several LAMP tests have been commercialized, [16], [21] and LAMP assays for tuberculosis, malaria, and HIV have been developed [20], [22]–[25].

There are several reasons why LAMP has had little impact on diagnostics designed for LRS. Foremost, LAMP-based NAATs still require electricity to achieve amplification temperatures (with an instrument, heat block, or water bath). Secondly, nonspecific amplification [20], [26]–[29] has been a challenge to assay development, and has only recently been addressed through sequence-specific detection strategies [20], [30]. To further advance the utility of LAMP and other isothermal technologies for LRS, this study builds upon previously published laboratory data [31]–[34] and supports the continued development of an electricity-free, self-contained platform that addresses these limitations.

Millions of lives and disability-adjusted life years are lost through delays in the correct and timely diagnosis of malaria, HIV, tuberculosis (TB), influenza, and other infectious diseases [7]. While our design is platform-based and therefore pathogen-agnostic, we chose to demonstrate this electricity-free molecular amplification and visual detection system using HIV-1 detection as a model analyte. Currently, no point-of-care (POC) molecular tests for HIV-1 exist in LRS, where detection of acute infections would have significant impact in high-disease-burden areas. Early identification and treatment of infected individuals could lower HIV transmission rates since acutely infective individuals are at a much higher risk of transmitting the virus [35]–[37]. Furthermore, the HIV-1 test could be used for POC early infant diagnosis, replacing conventional reference center testing, which has shown significant delays in reporting of results and loss to follow-up [38]. The experimental elements that have been integrated and tested here are intended to be combined with an electricity-free sample preparation technology (under development) to enable same day, same-visit testing and treatment of infants to greatly improve child health outcomes and substantially reduce loss to follow-up [39], [40].

Previously, we reported on the development of a non-instrumented nucleic acid amplification (NINA) platform [33] and we proposed an LRS-appropriate workflow [41] for an electricity-free kit. Since then, we have advanced the design from the proof-of-concept stage to an optimized, robust alpha prototype with significant improvements in performance and usability [42]. The improved NINA design we report on in this study requires fewer activation steps, and accordingly affords significantly less opportunity for user-introduced error and variation. Incorporating magnesium iron alloy (MgFe) into a diagnostic technology has been previously reported [43]. In the experimental device described here, replacing calcium oxide with magnesium iron alloy (MgFe) for the exothermic reaction offers several distinct advantages: (1) MgFe has significantly higher energy density, reducing the mass of the fuel pouch from 20 g to 1 g; (2) MgFe is commercially available at a very low cost (currently at US$.06 per test) with very little batch-to-batch variation; and (3) MgFe can be milled to a specific particle-size range to further control the heat profile of the chemical reaction. Additional improvements in this prototype include packaging the MgFe fuel in a hydrophilic, heat-sealable membrane and containing the liquid reactant in an easy-to-use blow-fill-seal container. These design enhancements greatly improve ease of use by minimizing user steps and eliminating the post-amplification heater cleaning that was required with previous designs. The improved design also incorporates a smaller vacuum-insulated housing to reduce heat loss and decrease the overall size of the heater. Finally, we incorporated an improved phase change material (PCM) with very high latent heat to meet the thermal requirements (Table 1) of an HIV-1 LAMP assay. To demonstrate the robustness of the improved heater design, we evaluated its thermal performance over a wide ambient temperature range that represents LRS conditions.

**Table 1:** Reverse transcription loop-mediated isothermal amplification (RT-LAMP) HIV-1 assay specifications.

Assay specifications	Value
Amplification temperature	61.5°C ±1.5°C
Amplification time	60 minutes
Sample warm-up time (ramp)	<2 minutes
Sample vessel	0.2 mL PCR tube

In addition to testing a new design and alternative heater materials, we further demonstrated the utility of the platform by incorporating a biplexed internal control as well as a nucleic acid lateral flow (NALF) visual detection method [44]. Multiplexed assays are commonly used in PCR for the detection of an internal control to confirm absence of inhibition. However, only a limited number of studies have examined multiplexed LAMP, [45]–[47] and to our knowledge, none have used NALF as the detection method. To demonstrate the utility of this evolving platform, we paired the technology with a NALF-detection cassette and evaluated the performance of the combined components using a biplexed LAMP assay for the detection of HIV-1 and a ß-actin internal control. Performance of the assay in the non-instrumented system was compared to parallel assays assessed in real-time on a thermocyler, with the sensitivity, reproducibility, and repeatability of the assay evaluated via melt curve analysis, NALF detection, and agarose gel electrophoresis of reaction products.

## Materials and Methods

### Electricity-free isothermal heater

**Figure 1:**
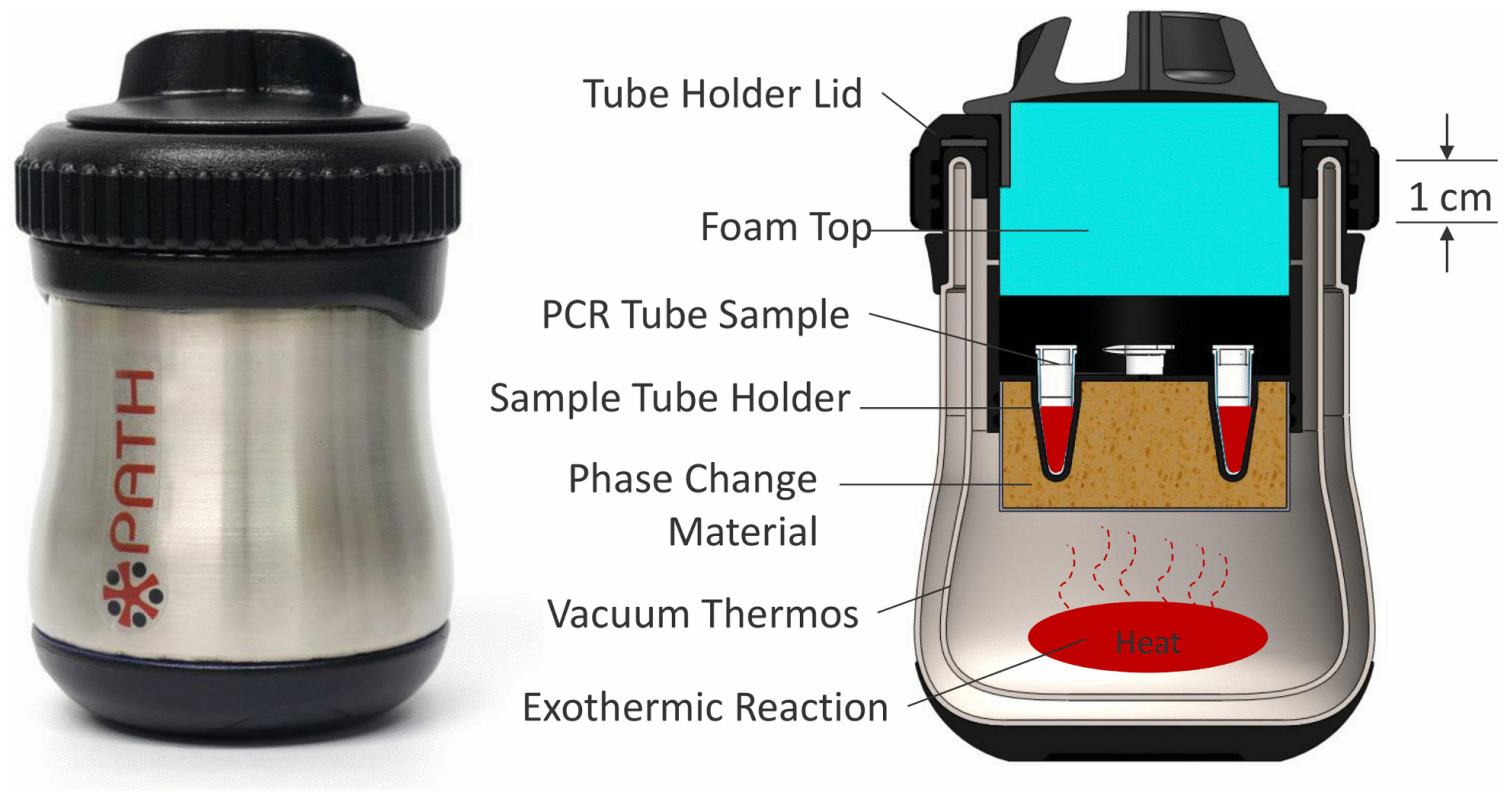
PATH NINA heater. (Right) Cross-section showing the internal components of the heater. Approximate dimensions of the heater are 80 mm diameter by 120 mm height.

Heaters were designed to heat the LAMP assay mixture in a 0.2 mL PCR tube to a temperature of 61.5°C +/−1.5°C for 60 minutes. The commercially available, double-walled, vacuum-insulated thermos (BS500BL003, Thermos, USA) shown in Figure 1 was used for the device housing due to its durability and insulating properties. The PCM, palmitic acid (27734-1KG, Sigma Aldrich, USA), is contained in a cup machined from highly conductive T-6061 alloy aluminum. To improve heat transfer, highly heat conductive, fine-stranded aluminum wool (7364T91, McMaster-Carr, USA) was cut into 50 mm disks and placed inside the cup prior to filling the cup with melted PCM that solidifies at room temperature. The PCM-filled cup is then bonded to UV-cured polymer tube holder lid (RGD525, Stratasys, USA) with J-B Weld epoxy (JB Weld, USA). The tube holder lid was designed to fit four 0.2 mL PCR tubes (981005, Qiagen, USA). Above the sample wells is a cover made from polyvinyl-chloride (PVC) foam (9318K77, McMaster-Carr, USA). The tube holder lid threads onto the vacuum-insulated container to retain the exothermic reaction. The foam insulation presses into the lid to prevent heat loss through the top of the device. Steam and hydrogen gas byproducts of the exothermic reaction are vented through small holes in the lid to prevent pressure build inside of the vacuum thermos.

The NINA heater configuration shown in Figure 1. is designed to be reusable. “Fuel pouches” and 5 mL 0.9% saline doses packaged in disposable cartridge (HCS20039Z, Medline, USA) are the only consumables required for each run. The fuel pouches consist of 0.9 g of MgFe fuel (Innotech Products Ltd., USA) sealed inside a hydrophilic heat-sealable pouch made from manila hemp, cellulose, and thermoplastic fibers (Empty Fillable Tea Bags, Muslin Bag, USA). The exothermic reaction produces heat through magnesium oxidation in water accelerated by galvanic corrosion [34]. Upon activation, the exothermic reaction occurs in the bottom of the thermos housing and heat is transferred to the PCM. The fuel pouches tested in this research were engineered to eliminate the need to weigh fuel prior to each test cycle and to facilitate the removal of the exothermic product residue after use [33].

### Operation of the heater and reference

To activate the NINA heater, the user places the MgFe fuel pouch in the thermos bottom, removes the sealed cap from a blow-fill-seal cartridge, and empties the cartridge contents of 5 mL (+/−0.2 mL) saline, onto the pouch. The blow-fill-seal cartridge is a commercially available disposable saline reservoir with a “twist-off” style cap. The MgFe fuel immediately reacts with the saline and produces heat. The tube holder is threaded onto the thermos and the lid is then placed on top until the target temperature is reached. After approximately 12 minutes, when the heater has warmed to 61.5°C +/−1.5°C, an isothermal assay in 0.2 ml PCR tubes can be inserted into the sample tube holders. Initial assay testing showed a loss in sensitivity when the assay was slowly heated. Therefore, adding the tubes after the 12-minute pre-warming step is preferred to achieve higher sensitivity with this particular assay (Table 2). After 60 minutes in the target temperature range, the tubes are removed for downstream processing using one of three methods, described below. After the test run, the exothermal reaction products contained in the pouch are discarded, while the thermos, insulation, PCM, and tube holders can be reused many times following a cool-down period. The thermos is rinsed with water to remove residual salt from the sidewalls.

**Table 2:** Details of the primer sets used for reverse transcription loop-mediated isothermal amplification (RT-LAMP) including the primers tagged for nucleic acid lateral flow (NALF) detection.

Primer name	Sequence (5′–3′)
***Primers for the detection of HIV-1***	
Integrase F3	GGT AAG AGA TCA GGC TGA ACA TC
Integrase B3	GCT GGT CCT TTC CAA AGT GG
Integrase BIP	Biotin/AGT GCA GGG GAA AGA ATA GTA GAC CTG CTG TCC CTG TAA TAA ACC C
Integrase FIP	CCC CAA TCC CCC CTT TTC TTA GAC AGC AGT ACA AAT GGC A
Integrase Loop B	GCA ACA GAC ATA CAA ACT AAA G
Integrase Loop F	FITC/TTA AAA TTG TGG ATG AAT
***Primers for the detection of β-actin***	
β-actin B3	AGG CCA GGA AGG AGG GAG
β-actin F3	GGC ATC CTC ACC CTG AAG T
β-actin BIP	Biotin/TG ACC GAG GCC CCC CTG AAC CAC CAG AAG AGG TAG CGG
β-actin FIP	DIG/TCC TCG GGA GCC ACA CGC AGC ATC GTC ACC AAC TGG GAC
β-actin Loop B	CGC GAG AAG ATG ACC CAG G
β-actin Loop F	GGT GCC AGA TTT TCT CCA TGT C

The HIV and β-actin primer sets are modified for lateral flow detection by the addition of biotin, fluorescein isothiocyanate (FITC), and digoxigenin (DIG).

### Performance monitoring

The thermal performance of the enhanced NINA heater was evaluated using two Type T thermocouples with +/−0.5°C precision (5SRTC-TT-K-20-36, Omega Engineering, USA) and a data logger (NI 9211 National Instruments, USA). As a proxy of actual reaction temperature, one thermocouple was inserted through a 1.27 mm diameter hole in the lid of a 0.2 mL PCR tube. The tube was filled with 25 µL deionized (DI) water to mimic the intended reaction volume and the thermocouple was submerged in the DI water at the bottom of the tube. The second thermocouple was attached to the bottom of the aluminum cup to monitor the exothermic reaction temperature.

### Testing the range of ambient operating temperatures

To determine the ambient operating temperature range, performance of the NINA heaters was assessed at ambient temperatures of 12°C, 14°C, 16°C, 30°C, 31°C, and 32°C in an environmental chamber (BTl-433, ESPEC, USA). Three heaters with instrumented DI water proxy-samples, fuel pouch, and saline tube were placed in the chamber for four hours to condition the materials to the test temperature. The heater reactions were then activated inside the environmental chamber and thermal performance was monitored during the one-hour incubation period.

### Sample preparation

Normal human plasma (NHP) containing HIV-1 virions (AcroMetrix HIV-1 High Control, Applied Biosystems, USA) was used as an HIV-1 RNA standard for assessing the performance of the HIV-1 LAMP assay using the NINA heater. Viral RNA was extracted from 200 µL of the NHP using QIAamp viral RNA mini kits (Qiagen Inc, USA) according to the manufacturer’s instructions. RNA was eluted into 60 µL of RNase-free water. For tests using the β-actin assay, total DNA/RNA was extracted from 200 µL whole blood using the DNeasy Blood and Tissue Kit (Qiagen Inc, USA) and eluted into 100 µL nuclease-free water. RNA was stored at −80°C until needed. DNA/RNA was quantified using a Nanodrop 2000 (Thermo Scientific, USA). Viral copy number was determined using the listed virion count and the dilution factor with an assumption of 100% extraction efficiency. Using the quantified copy number, serial dilutions were prepared in RNase-free water. Negative control reactions used the same volume of RNA eluate extracted from HIV-negative NHP.

### LAMP primer design

Primers targeting the *pol* integrase gene region as described by Hosaka *et al.* [32] were used to detect HIV-1 (Table 2) with two primers modified by the addition of either fluorescein isothiocyanate (FITC) or biotin to facilitate detection via lateral flow strips. After testing different combinations of modified loop and inner primers, a FITC-labeled backward inner primer (BIP) primer and a biotin-labeled Loop F primer were selected for the HIV assay (Table 2). As an internal amplification control, a primer set for the detection of human β-actin mRNA was designed using Primer Explorer V4 software for LAMP primer design (Eiken Chemical Co, Japan) with β-actin BIP and β-actin forward inner primer (FIP) tagged with biotin and digoxigenin (DIG) respectively (Table 2).

### HIV RT-LAMP assay conditions

Amplification was performed in 25 µL reactions using individual 0.2 mL flat lid PCR tubes (Qiagen, Germany). Each reaction contained 2× Reaction Mix from the LoopAmp DNA Amplification Kit (Eiken, Japan), 0.2 µM intergrase F3, 0.2 µM intergrase B3, 1.6 µM intergrase BIP biotin, 1.6 µM intergrase FIP, 1.6 µM intergrase Loop B, 1.6 µM intergrase Loop F FITC, 2U avian myeloblastosis virus (AMV) reverse transcriptase (New England Biolabs, USA), and 16 U Bst DNA polymerase large fragment (Eiken, Japan). For detection of DNA amplification in real-time, the intercalatory dye SYTO-85 (Life Technologies, USA) was added to a final concentration of 1 µM. HIV RNA at final concentrations of 19, 38, 75, and 150 copies/reaction were added to the reaction tubes prior to their placement into the NINA heater. A Negative control of RNA from non-infected NHP were also included. Each HIV dilution, as well as the negative control was screened in 21 replicate reactions. To prevent evaporation, 15 µL of mineral oil was added to the top of each reaction prior to incubation. Reaction tubes were added to the NINA heater after the 12-minute warm-up. Duplicate tests were run in parallel using the NINA heater as well as the Stratagene MX3000P Real-Time PCR system (henceforth, the Stratagene: Stratagene, USA – superseded by the Mx3000P QPRC from Agilent Technologies, USA, www.genomics.agilent.com). The Stratagene samples were amplified at 62°C for 60 minutes and monitored for DNA amplification in real-time via an intercalatory fluorescent dye (SYTO 85, Molecular Probes, USA) every 60 seconds.

### Modifications to HIV reverse transcription loop-mediated isothermal amplification (RT-LAMP) assay for use in isothermal heater

**Figure 2:**
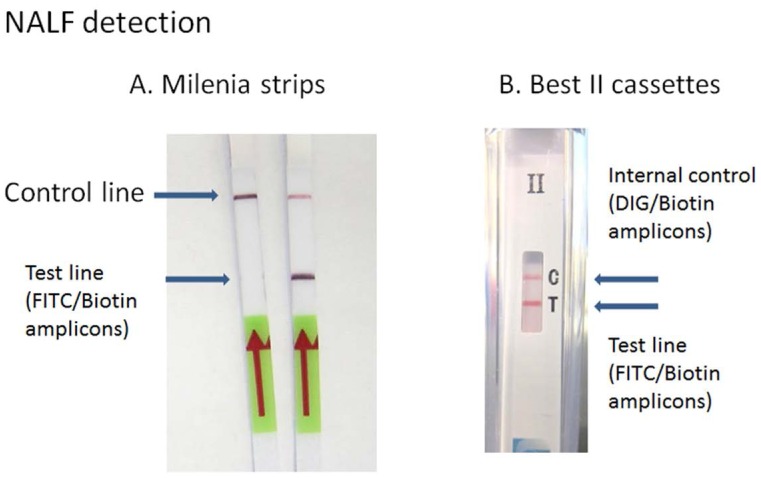
HIV LAMP amplicon detection via Milenia test strips. (A) Milenia strips used for the detection of fluorescein isothiocyanate (FITC)/biotin-labeled amplicons. Image to the right shows a positive result while image to the left is a negative result. (B) Best cassettes for the dual detection of FITC/biotin (HIV assay) and digoxigenin (DIG)/biotin (β-actin assay)–labeled amplicons. Image shows a positive result for both FITC/biotin amplicons and DIG/biotin amplicons.

To demonstrate electricity-free amplification and detection suitable for POC diagnosis, we used NALF for visual detection of the amplified product. To accomplish this, two primers in each target were modified by hapten labeling to enable capture via antibody binding of FITC and subsequent visual detection of captured amplicons via streptavidin colloidal gold. HIV LAMP assays with labeled and unlabeled primers were run in parallel and their performance was measured in real-time. Hapten labeling of the BIP and LOOP F primers had no effect on the performance of the HIV assay. Furthermore, the labeled primers enabled HIV LAMP amplicon detection via Milenia lateral flow test strips (Milenia Biotec Gieben, Germany) (Figure 2).

### HIV and β-actin biplexed LAMP

Amplification was carried out as previously described for the HIV-1 assay except that primers for the detection of β-actin and HIV were included at the following concentrations: 0.1 µM β-actin B3, 0.1 µM β-actin F3, 0.8 µM β-actin BIP, 0.8 µM β-actin FIP, 0.4 µM β-actin Loop B, 0.4 µM β-actin Loop F, 0.1 µM F3, 0.1 µM B3, 0.8 µM BIP biotin, 0.8 µM FIP, 0.8 µM Loop B, 0.8 µM Loop F-FITC. RNA was added to each reaction to give a final concentration of 73, 145, 290, and 580 copies/reaction respectively. With each set of dilutions, three replicates were assessed in the presence and absence of 2.2 ng/µL genomic DNA. Negative controls were also included. Mineral oil in aliquots of 15 µL was added to each tube to prevent evaporation during incubation. Reactions were heated both in the electricity-free NINA heater and the real-time PCR machine. Positive results were scored as those detected by NALF, and results were confirmed using melt curve analysis and gel electrophoresis.

### Assessment of LAMP amplicons

Performance of the NINA heater as measured by limit of detection (LOD) was compared to the Stratagene real-time thermocycler. Replicates of 21 samples containing 19, 38, 75, and 150 HIV RNA copies/reaction were amplified using the HIV-1 RT-LAMP assay in both the NINA heaters and the Stratagene real-time thermocycler. Positives were scored as those detected by NALF, with results confirmed using melt curve analysis and gel electrophoresis (data not shown). In addition, real-time detection was also carried out on the samples amplified using the Stratagene real-time thermocycler (Table 2).

#### Nucleic acid lateral flow detection

**Figure 3:**
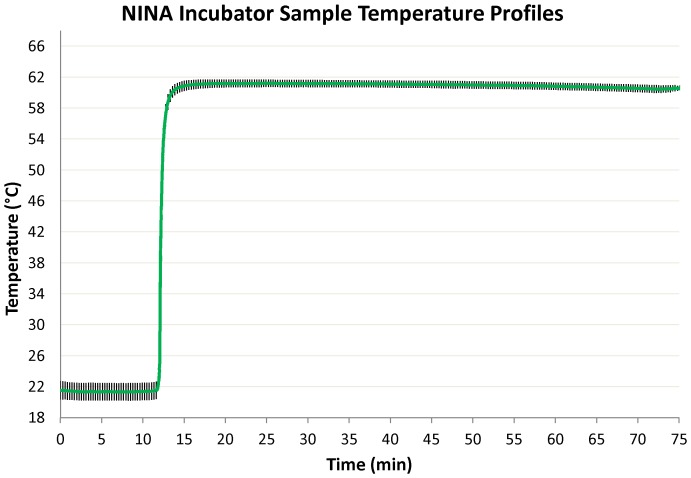
Average temperature profiles of 74 runs with nine prototype non-instrumented nucleic acid amplification (NINA) heaters. Error bars show one standard deviation. Four devices were removed from testing when a failure mode rendered them no longer able to maintain temperature within specification. Final run data are not included in the graph. Mean time between failures (MTBF) for the failed devices is 14 runs.

Detection of the labeled amplicons was assessed using HybriDetect dipsticks (Milenia Biotec Gieben, Germany) for FITC/biotin amplicons, or the BioHelix Express Strip (BESt) cassettes Type II (Biohelix, USA) for dual detection of FITC/biotin and DIG/biotin amplicons (Figure 3). Prior to introducing the amplified product to the detection strips, the RT-LAMP reactions were terminated by the addition of 1.5 µL EDTA (100 mM; Cellgro, USA) to 13.5 µL of the reaction.

For the Milenia strips, 5 µL of the terminated reaction was placed on the capture pad followed by immediate immersion of the pad into 100 µL of 1× HybriDetect assay running buffer. Test results were scored after 5 minutes. A positive result was indicated by visible test and control lines, and a negative result was indicated by a visible control line only. Tests were considered invalid if the upper control line did not develop. For the BESt cassettes, 7.5 µL of each reaction was removed from each tube for melt curve analysis. The PCR tube containing the remaining reaction was then placed into the BESt cassette. The cassette was closed according to the manufacturer’s instructions and tests were scored after 5 to 10 minutes with a positive result demonstrated by the presence of the band on the strip for either FITC/biotin detection and/or DIG/biotin detection.

#### Melt curve analysis

After NALF analysis, the remaining 7.5 µL of each amplified reaction mixture from both the Stratgene real-time thermocycler and the NINA heater were analyzed via melt curve analysis to further confirm that the correct amplicons had been generated in the RT-LAMP reactions. Samples were placed in the Stratagene MX3000P Real-Time PCR system and were heated from 54°C to 94°C at a ramp rate of 1°C/s. Fluorescence data was collected and the first derivate was plotted against temperature to create a unique peak for each amplicon indicating its melt temperature (T_m_). The observed T_m_ was compared to the T_m_ for the expected amplicon product as a check for the specificity of amplification reactions. Unexpected or multiple peaks were considered evidence of non-specific amplification and/or contamination, and the suspect reactions were considered false positives if a negative result was observed using NALF.

#### Agarose gel electrophoresis

Each reaction product was also analyzed on a 2% agarose gel (Invitrogen, USA) that had been stained with 1× SYBR Safe (Invitrogen, USA) gels and were run in 1× TBE running buffer and visualized under UV light. A 1 Kb plus ladder (Invitrogen, USA) was included as an amplicon size marker. Gels were examined for the presence of the banding pattern indicating the amplicon polydispersity characteristic of a LAMP reaction. Absence of the characteristic pattern was considered evidence of non-specific amplification, and the suspect reactions were considered false positives if a negative result was observed using NALF (Data not shown).

## Results

### NINA heater testing

For the laboratory testing, nine NINA heaters were evaluated for a total of 74 runs. Results demonstrate highly precise temperature control with one standard deviation between 60°C and 62°C for the entire hour for those devices that did not undergo mechanical failure (Figure 3).

As part of the evaluation, each NINA heater was run multiple times. These runs were logged and a device was removed from further evaluation if, after multiple successful runs, it suddenly failed to perform to the required 61.5°C +/−1.5°C specification. An investigation was then conducted to determine the failure mode that caused the out-of-specification condition. Of the nine identical prototypes fabricated and used in assay testing, four failed to maintain temperature for the specified time after repeated use (mean time to failure was 14.25 runs, range was 5 to 29 runs). The most common cause of these specification failures was a failure of the epoxy bond between the PCM container and lid.

**Figure 4:**
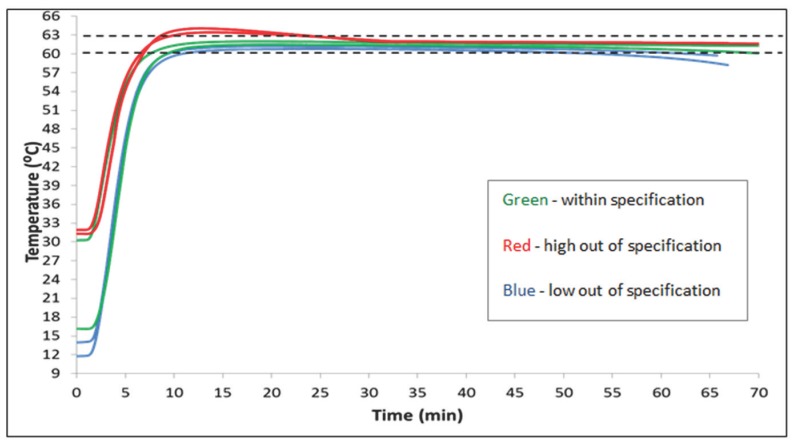
Three non-instrumented nucleic acid amplification (NINA) heaters were conditioned in an environmental chamber, and the performance of each heater was determined at multiple temperatures (12°C, 14°C, 16°C, 30°C, 31°C, and 32°C). Averages are shown. The green lines show averages that were within the specification of 61.5°C +/−1.5°C. The red lines show runs that over-heated, and the blue lines show runs that fell below the test specifications.

To evaluate the ambient temperature operating range, we measured tube sample temperature inside the conditioned NINA heater from time zero. The results in Figure 4 show an operationally acceptable ambient temperature range for the NINA heater to be between 16°C and 30°C.

### Preliminary testing with the LAMP assay

Preliminary testing and analysis was performed to determine the effects of placing the assay tube in the heater before the heater achieved the targeted incubation temperature. A series of HIV LAMP assays challenged with 150, 75, 38, and 17 copies of HIV RNA were prepared in triplicate in addition to negative controls. We measured test performance using melt curve analysis of completed assays, in addition to lateral flow detection and gel electrophoresis. Results are shown in Table 3. Introducing the test reactions into the heater prior to the warm-up had a negative effect on sensitivity with the LOD being 150 HIV-1 copies/reaction as compared to 38 HIV-1 copies/reaction if the reactions were added after 12 minutes (Table 3). The addition of mineral oil was found to have no negative effect on assay performance with 3/3 replicates producing positive results at 38 HIV-1 copies/reaction compared with 2/3 replicates for assays run without the addition of oil. Neither the warm-up ramp nor mineral oil affected specificity as all human plasma controls in the preliminary testing were negative.

**Table 3:** A comparison of the effects on the limit of detection (LOD) of assays heated by the non-instrumented nucleic acid amplification (NINA) heater.

	No oil/ramp	Oil/ramp	No oil/no ramp	Oil/no ramp
**150 copies/reaction**	3/3	3/3	3/3	3/3
**75 copies/reaction**	1/3	2/3	3/3	3/3
**38 copies/reaction**	0/3	0/3	1/3	3/3
**19 copies/reaction**	0/3	0/3	2/3	2/3
**Negative control**	0/3	0/3	0/3	0/3

Combination of assays run with and without warm-up ramp and with or without oil are evaluated. Table entries show the number of replicates that returned a positive result as the numerator of a fraction showing the total number of replicates run in the denominator.

### Evaluation of LAMP reactions performed in the NINA heater

We compared assay performance of the electricity-free NINA heater and NALF detection systems to using a real-time PCR machine. Results using either heater were very similar, with an LOD of 75 copies/reaction or 8,333 viral copies/mL of extracted plasma observed for both incubation methods (Table 4). Melt curve analysis of each reaction further confirmed the amplification of the correct products with average melting temperature of ∼78°C observed for all positive reactions across the dilution series in both the NINA heater and the real-time thermocycler.

**Table 4:** An evaluation of the non-instrumented nucleic acid amplification (NINA) heaters as compared to a real-time PCR thermocycler by measuring the performance of the HIV LAMP assay with a range of HIV RNA target concentrations.

	NINA incubator	Thermocycler
**150 copies/reaction**	21/21	21/21
**75 copies/reaction**	21/21	20/21
**38 copies/reaction**	18/21	16/21
**19 copies/reaction**	17/21	13/21
**Negative control**	0/21	0/21

All results were confirmed by NALF, agarose gel electrophoresis, and melt curve analysis. Table entries show the number of replicates that returned a positive result as the numerator of a fraction showing the total number of replicates run in the denominator.

### Biplexed LAMP performance in the NINA heater

**Figure 5:**
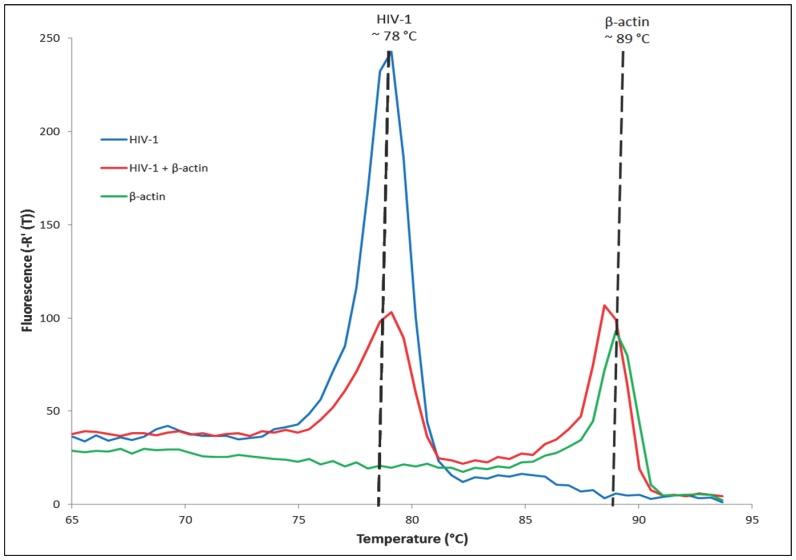
Melt curve analysis of the HIV-1 and β-actin biplex reverse transcription loop-mediated isothermal amplification (RT-LAMP) assay. Melt analysis showed products specific to HIV-1 at 78°C and β-actin at 89°C as seen in the singleplex assays. Specificity was confirmed in the presence of normal human plasma (NHP) without HIV-1 or β-actin (negative control) and showed no amplification (n = 3).

Initial tests with 2.2 ng/reaction of genomic DNA and RNA containing β-actin mRNA in the β-actin LAMP assay confirmed rapid detection of β-actin RNA using both real-time analysis and NALF detection. Similar results were observed when using both NINA and Stratagene heating (Table 5). Amplification of human β-actin was observed in every reaction. Melt curve analysis indicated that the amplicons of the HIV-1 LAMP assay and the β-actin assay produced unique peaks at 78°C and 89°C, respectively (Figure 5). The presence and absence of a peak generally mirrored the results observed with NALF detection, but the inclusion of the β-actin primer set was found to decrease the sensitivity of HIV-1 detection (Table 5).

**Table 5:** Results for normal human plasma (NHP) containing HIV-1 RNA diluted to 580, 290, 145, 73, and 0 (negative control) total copies in a 25 µl reaction.

	Strategene		Heater device	
Sample	HIV-1 positive/total	β-actin positive/total	HIV-1 positive/total	β-actin positive/total
**580 copies HIV-1+ β-actin**	3/3 (100%)	3/3 (100%)	3/3 (100%)	3/3 (100%)
**290 copies HIV-1+ β-actin**	3/3 (100%)	3/3 (100%)	2/3 (100%)	3/3 (100%)
**145 copies HIV-1+ β-actin**	2/3 (100%)	3/3 (100%)	1/3 (100%)	3/3 (100%)
**73 copies HIV-1+ β-actin**	1/3 (33%)	3/3 (100%)	0/3 (0%)	3/3 (100%)
**Negative control**	0/3 (0%)	0/3 (0%)	0/3 (0%)	0/3 (100%)
**β-actin positive control**	0/3 (0%)	3/3 (100%)	0/3 (0%)	3/3 (100%)
**HIV-1 positive control (580 copies)**	3/3 (100%)	0/3 (0%)	3/3 (100%)	0/3 (0%)

Extracted genomic nucleic acid at 2.2 ng/µL was in all samples except the negative control and HIV-positive control. In this comparison, samples and controls assayed by the non-instrumented nucleic acid amplification (NINA)/nucleic acid lateral flow (NALF) system and the Stratagene system were aliquoted from the same sample extraction and handled identically up to amplification. Since the NINA heater does not have top-heating, oil was added to these samples for amplification to control for any condensation artifacts. The samples in the top-heated Stratagene were amplified as per the manufacturer’s instructions (no mineral oil) to reflect a standardized reference method.

## Discussion

While other electricity-free heaters have been proposed, [43], [48], [49] these designs have relied heavily on the use of passive cooling strategies, and their performance has not been demonstrated outside a narrow range of standard laboratory temperatures. In this study, we demonstrate the performance of an improved electricity-free heater over a wide temperature range (16°C to 30°C) that can be expected in the low-resource settings where we seek to deploy molecular-based diagnostics. However, the electricity-free heater is only an enabling technology, not a complete solution. Thus, to further demonstrate the potential for diagnosis based on electricity-free nucleic acid amplification, we have paired the NINA heater with other complementary, instrument-free technologies, such as NALF, to demonstrate enabling technology compatibility toward building a completely infrastructure-independent system. To illustrate the utility of the electricity-free NINA heater and NALF detection platform, we demonstrate sensitive and repeatable detection of HIV-1 and ß-actin from an extracted sample to result in less than 80 minutes.

One remaining need, not addressed by the research presented here, is the development of an appropriate sample preparation method. Current methods are generally costly and require dedicated equipment and reagents as well as skilled technicians. A key advantage of LAMP is its tolerance to many confounders, including whole blood products, [50] which indicates that more crude sample preparation methods than those required for PCR could be used. Successful detection of the target amplicon has been demonstrated by adding whole blood directly to LAMP assays, thus eliminating the need for any sample preparation [20]. A complete electricity-free NAAT-based diagnostic solution will probably require incorporation of shear-based lysis techniques, [51], [52] the filtering and concentrating gained by FTA cards, [53] an electricity-free centrifuge, [54], [55] or a simplified chaotroic salt extraction methodology that requires no centrifuge [56].

Inclusion of an endogenous control primer set decreased sensitivity for the HIV-1 target. Other studies have also noted decreased single assay performance in biplexed LAMP reactions, with one study demonstrating a negative effect on time to result [47]. In this study, since the β-actin mRNA and DNA were more concentrated than the HIV-1 RNA, they may amplify more effectively and, therefore, restrict the HIV-1 assays’ sensitivity by sequestering the limited reagents for reverse transcription and DNA amplification. Further optimization of the biplex reaction is expected to improve upon these results.

There are many barriers to the use of highly accurate molecular tests in the infrastructure-limited settings found at the POC in low-resource regions of the world. Among these are unreliable electricity, minimally trained users, and extreme ambient temperatures. By combining the improved NINA heater design with a biplexed isothermal assay and endpoint detection by NALF, we demonstrated the synergistic potential of these state-of-the-art technologies while also identifying challenges and scope for future development. The diagnostic system described in this paper reliably detects HIV-1 RNA and the endogenous control in prepared samples within 80 minutes. These key elements of an easy-to-use, low-cost, flexible platform design can be adapted for use with many isothermal NAATs for diagnosis of a variety of pathogens where molecular detection is preferred. When the components demonstrated herein are paired with electricity-free sample preparation, the complete system may represent an alternative to the currently available molecular diagnostic testing methods. We envision the NINA platform enabling case detection and surveillance well beyond centralized laboratories, at lower levels of the health care system where infrastructure and training cannot support currently available molecular methods that require instrumentation and electricity.
